# Cerebral Air Embolism After Navigational Bronchoscopy Treated With Aspiration Thrombectomy: A Case Report

**DOI:** 10.7759/cureus.117776

**Published:** 2026-10-11

**Authors:** Matthew Eardley, Anushka Jayakrishnan, Essam Hashem, Darshana Dhaka, Ashvini Menon

**Affiliations:** 1 Thoracic Surgery, University Hospitals Birmingham NHS Foundation Trust, Birmingham, GBR; 2 Neuroradiology, University Hospitals Birmingham NHS Foundation Trust, Birmingham, GBR; 3 Anaesthesiology, University Hospitals Birmingham NHS Foundation Trust, Birmingham, GBR

**Keywords:** aspiration thrombectomy, cerebral air embolism, large-vessel occlusion, mechanical thrombectomy, navigational bronchoscopy, systemic arterial air embolism, transbronchial biopsy

## Abstract

Systemic arterial air embolism is a rare but potentially devastating complication of bronchoscopic lung biopsy. A 68-year-old man with severe emphysema developed abrupt bradycardia, hypotension, and failure to regain consciousness after navigational bronchoscopy under general anaesthesia. Computed tomography demonstrated air within the left middle cerebral artery, and angiography confirmed proximal large-vessel occlusion. Urgent aspiration thrombectomy achieved complete reperfusion after one pass, with retrieved material compatible with thrombus. Despite technically successful reperfusion, major neurological deficits persisted. This case highlights haemodynamic collapse and delayed emergence as warning signs and supports rapid neuroimaging with selective endovascular treatment when an accessible large-vessel occlusion is present.

## Introduction

Systemic arterial gas embolism is a rare but potentially devastating complication of bronchoscopic lung biopsy, with a reported incidence of less than 0.02% during flexible bronchoscopy [1]. In a systematic review of 29 reported cases following bronchoscopic lung biopsy, mortality was 28%, residual neurological deficits occurred in 24%, and 48% of patients achieved full recovery [1]. Air entry is thought to occur when disruption of an airway, alveolar, or bronchovascular interface permits communication with the pulmonary venous circulation and a favourable pressure gradient facilitates systemic embolisation; positive-pressure ventilation may contribute to this process [1-3].

Clinical presentation may include acute neurological or cardiac deterioration, abrupt haemodynamic instability, or delayed emergence from anaesthesia [1]. Sudden bradycardia and hypotension occurring during or shortly after biopsy may therefore provide an early clinical clue, although these findings are not specific for cerebral air embolism. When neurological deterioration or unexplained delayed emergence occurs, urgent computed tomography (CT), with CT angiography when appropriate, can identify intracranial air and determine whether a large-vessel occlusion is present. Initial management includes high-concentration oxygen and consideration of hyperbaric oxygen therapy [4,5]. Endovascular aspiration is not routine treatment for cerebral air embolism but has been reported in selected patients with an accessible large-vessel occlusion [6]. Cerebral arterial air embolism has previously been described following navigational bronchoscopy [7], while more recent reports during robotic-assisted bronchoscopy demonstrate that advanced peripheral biopsy platforms do not eliminate this risk [8].

## Case presentation

A 68-year-old man with alpha-1 antitrypsin deficiency-associated emphysema and previous thoracotomies underwent elective navigational bronchoscopy with transbronchial biopsy of a right lower lobe pulmonary lesion. Pre-procedural computed tomography (CT) demonstrated bullous lung disease on the lung-window image (Figure 1A) and a peripheral lesion abutting the right inferior pulmonary vein on the soft-tissue window (Figure 1B). At multidisciplinary review, positron emission tomography-CT was not considered necessary, but tissue diagnosis was required to distinguish relapse of known mucosa-associated lymphoid tissue lymphoma from a new primary lung malignancy. Previous conventional bronchoscopy had not achieved tissue diagnosis. The patient was reluctant to undergo surgical biopsy, and navigational bronchoscopy was selected as a less invasive approach. The lesion was not considered difficult to localise.

**Figure 1:**
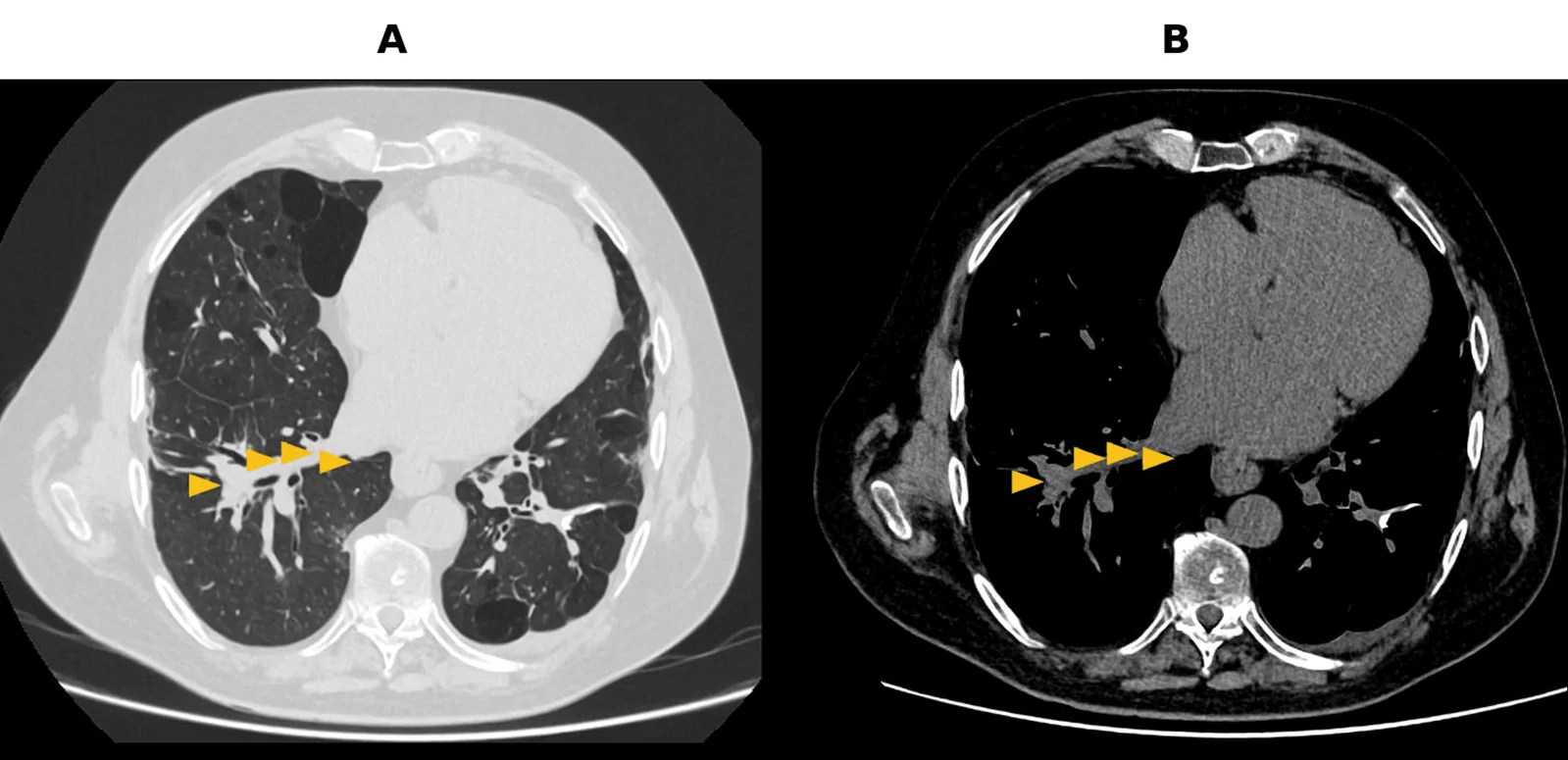
Pre-procedural thoracic computed tomography (A) Axial lung-window image demonstrating bullous emphysematous lung disease and the peripheral right lower lobe target lesion. (B) Axial soft-tissue window demonstrating the target lesion abutting the right inferior pulmonary vein. Yellow markers indicate the lesion and adjacent pulmonary vein. CT = computed tomography.

Bronchoscopy was performed under general anaesthesia with total intravenous anaesthesia, endotracheal intubation, and mechanical ventilation. Positive end-expiratory pressure was maintained at 8-10 cm H2O, and recruitment manoeuvres were performed with airway pressure limited to 25 cm H2O. Five biopsy passes were performed as routine practice for tissue adequacy. After the fifth and final pass, mild bleeding from the right lower lobe bronchus was controlled with cold saline lavage. At approximately 10:25, during lavage, the patient developed abrupt bradycardia to approximately 38 beats/min and hypotension to approximately 58/32 mmHg with a weak carotid pulse. Atropine, ephedrine, and intravenous crystalloid produced haemodynamic improvement. He remained supine during initial stabilisation. The acute sequence of events is summarised in Table 1.

**Table 1:** Acute event timeline CT = computed tomography; GCS = Glasgow coma scale; TICI = thrombolysis in cerebral infarction

Time	Event
9:15	Induction of general anaesthesia and endotracheal intubation
9:30	Navigational bronchoscopy commenced
10:24	Final biopsy pass completed; mild bleeding noted from right lower lobe bronchus
10:25	Cold saline lavage performed for haemostasis; sudden haemodynamic collapse during lavage (heart rate ~38 beats/min, blood pressure ~58/32 mmHg, weak carotid pulse)
10:26	Atropine 0.6 mg, ephedrine 12 mg, and 500 mL Hartmann's solution administered
10:30	Attempted emergence from anaesthesia; sugammadex 600 mg, doxapram 20 mg x2, and naloxone 200 micrograms administered
10:30-11:00	Patient remained unresponsive (GCS E1 V1 M1)
11:00	Stroke consultant contacted; decision for urgent neuroimaging
11:38	Urgent CT brain and CT angiography requested
12:12	CT brain and CT angiography performed
12:34-12:36	Stroke team reviewed imaging; accepted by neuroradiology for emergency mechanical thrombectomy
13:00	Patient arrived in angiography department
13:20	Procedure started with right common femoral artery puncture
13:36	First and final aspiration pass performed; retrieved material sent for histology
13:50	Final angiographic run completed; TICI 3 reperfusion achieved
14:15	Returned to the theatre recovery area, intubated
19:40	Admitted to the intensive care unit

Despite pharmacological reversal of anaesthesia, the patient remained unresponsive with a Glasgow Coma Scale score of E1 V1 M1. Urgent neuroimaging was arranged after discussion with neurology. CT and CT angiography were performed at 12:12, approximately 1 hour 47 minutes after the haemodynamic collapse. Non-contrast CT demonstrated hypodense intravascular air within the left middle cerebral artery (Figure 2A) and adjacent hyperdense intraluminal material consistent with thrombus (Figure 2B). CT angiography demonstrated restriction of the left middle cerebral artery lumen and confirmed a proximal large-vessel occlusion (Figure 2C). These findings raised concern for a mixed air-thrombotic occlusion. Hyperbaric oxygen therapy was not available locally; the nearest hyperbaric centre was more than one hour away, and no established emergency transfer pathway existed. Given the discrete, accessible proximal arterial occlusion, the patient was accepted immediately for emergency mechanical thrombectomy.

**Figure 2:**
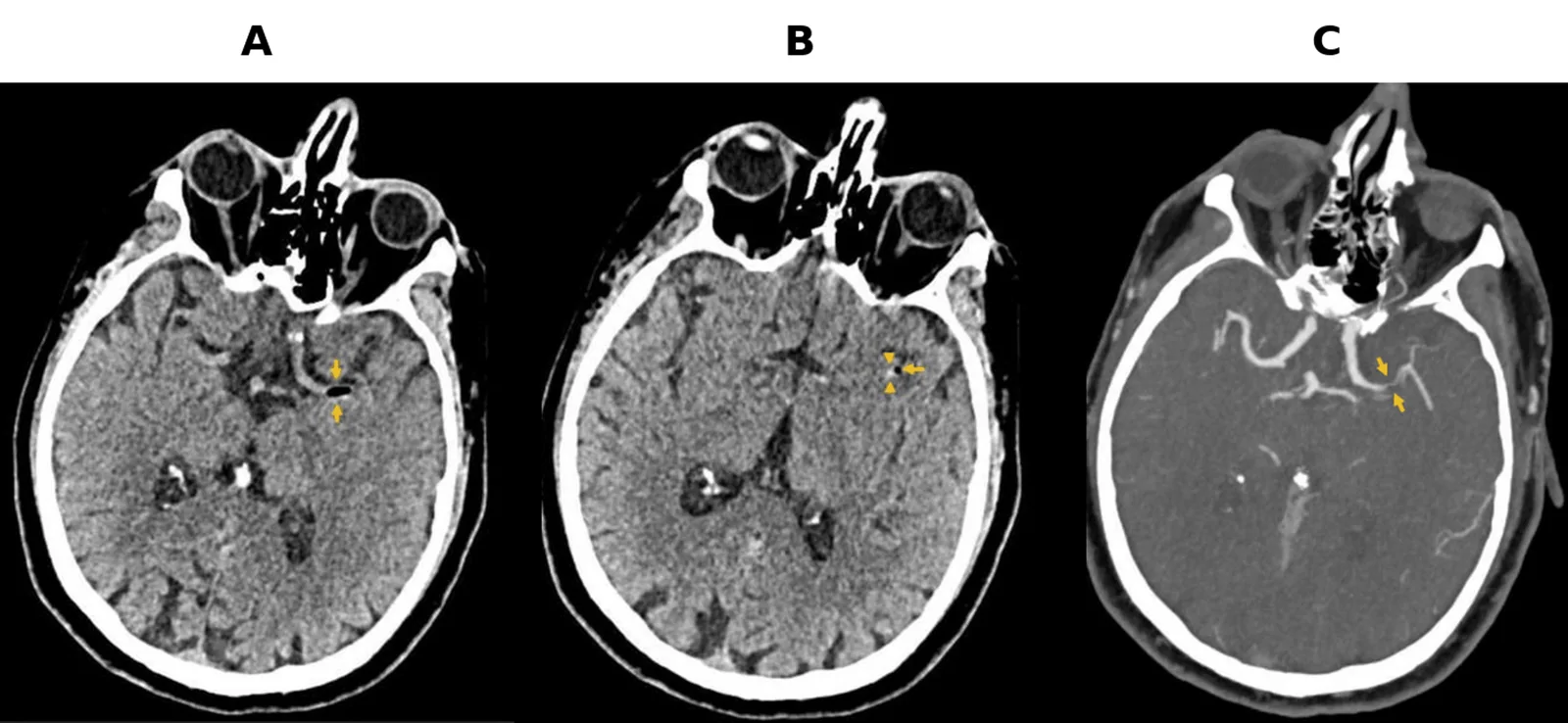
Neuroimaging following failure to regain consciousness (A) Non-contrast computed tomography demonstrating hypodense intravascular air within the left middle cerebral artery (arrows). (B) Non-contrast computed tomography demonstrating adjacent hyperdense thrombotic material (arrowheads) and hypodense air (arrow). (C) Computed tomography angiography, axial maximum-intensity projection, demonstrating restriction of the left middle cerebral artery lumen associated with air embolism and thrombus (arrows). CT = computed tomography; CTA = computed tomography angiography; MCA = middle cerebral artery

Mechanical thrombectomy was performed through transfemoral arterial access using a Sofia Plus 6F aspiration catheter (Terumo Neuro, Aliso Viejo, California, USA). Pre-treatment digital subtraction angiography demonstrated filling defects within the left middle cerebral artery (Figure 3A). A single aspiration pass was performed at 13:36, and the final angiographic run at 13:50 demonstrated Thrombolysis in Cerebral Infarction grade 3 reperfusion (Figure 3B). This corresponded to approximately 3 hours 25 minutes from haemodynamic collapse and 3 hours 20 minutes from failure to regain consciousness to complete reperfusion. Retrieved material was histologically compatible with thrombus, supporting the presence of a thrombotic component within the occluded left middle cerebral artery rather than isolated intravascular air alone.

**Figure 3:**
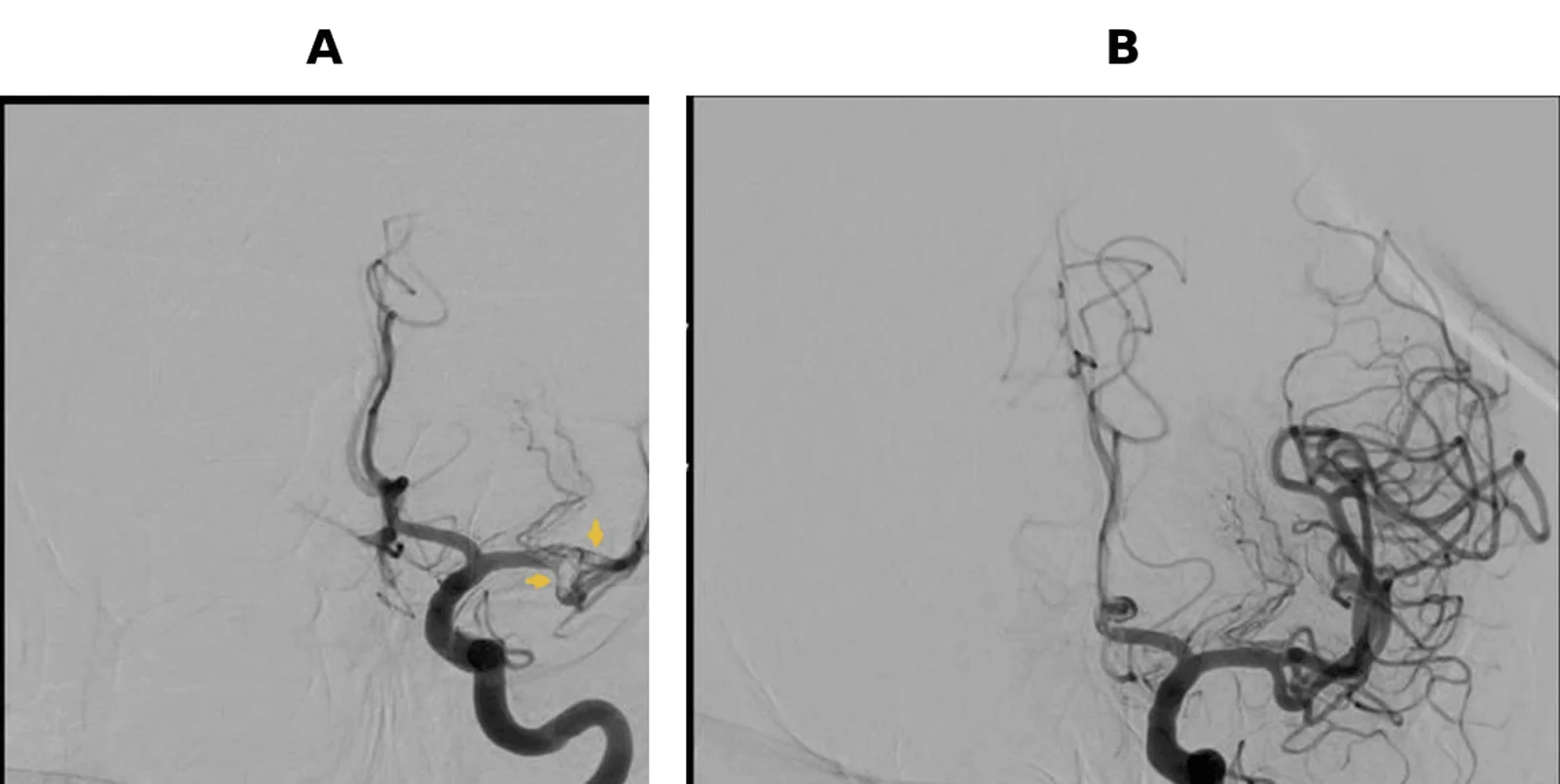
Digital subtraction angiography before and after aspiration thrombectomy (A) Pre-treatment left internal carotid artery angiogram in Towne projection demonstrating filling defects within the left middle cerebral artery, compatible with air embolism and thrombus (arrows). (B) Post-aspiration angiogram demonstrating resolution of the filling defects and restoration of flow to the left middle cerebral artery territory, with TICI 3 reperfusion. DSA = digital subtraction angiography; ICA = internal carotid artery; MCA = middle cerebral artery; TICI = thrombolysis in cerebral infarction

The patient subsequently required intensive care management. Despite complete angiographic reperfusion, neurological recovery was limited. At 20 days, he had persistent global aphasia, dysphagia, and dense right-sided weakness and was discharged to inpatient stroke rehabilitation.

Written informed consent for open-access publication was obtained from the patient’s next of kin.

## Discussion

The key diagnostic challenge in this case was distinguishing a rare procedural complication from delayed emergence after general anaesthesia. Several case-specific features may have increased susceptibility to systemic embolisation, including severe emphysema, previous thoracic surgery, a peripheral lesion abutting the right inferior pulmonary vein, repeated transbronchial sampling, airway bleeding after the final biopsy, positive end-expiratory pressure, and recruitment manoeuvres. Together, these findings support a proposed mechanism involving transient communication between an injured airway or alveolar surface and the pulmonary venous circulation, with a pressure gradient that may facilitate air entry [1-3]. However, no direct broncho-venous communication was demonstrated. The anatomical proximity of the target lesion to a pulmonary vein may represent a potential procedural risk factor, but its contribution to the development of air embolism cannot be established from this single case.

Abrupt haemodynamic deterioration may provide an early clinical clue. Bradycardia and hypotension have been reported in bronchoscopic air embolism cases and may be particularly concerning when they occur abruptly during or immediately after biopsy [1,9]. In this patient, the sudden fall in heart rate and blood pressure occurred immediately after the final biopsy and lavage, before failure to regain consciousness became apparent. Although coronary air embolism is one possible cause of haemodynamic collapse, no electrocardiographic evidence of acute coronary ischaemia was identified in this case; therefore, the precise mechanism of the haemodynamic deterioration remains uncertain. These findings should not be considered specific or reliably predictive of cerebral air embolism in isolation.

Delayed emergence following bronchoscopic biopsy should not automatically be attributed to anaesthesia once routine anaesthetic causes have been addressed. Intravascular air may dissipate rapidly, making early CT particularly important [1,4]. In this case, CT demonstrated intracranial air, while CT angiography identified a discrete proximal left middle cerebral artery large-vessel occlusion. The additional finding of adjacent hyperdense intraluminal material and the retrieval of material histologically compatible with thrombus support the presence of a thrombotic component within the occluded artery. These findings are therefore more consistent with a mixed air-thrombotic occlusion than with isolated cerebral air embolism alone.

Initial management of cerebral arterial gas embolism includes cessation of the procedure, high-concentration oxygen, and appropriate positioning. For systemic arterial embolism arising from the pulmonary venous circulation, supine or right lateral positioning has been recommended rather than the left lateral Trendelenburg position traditionally used for venous air embolism [4,5]. Hyperbaric oxygen is an important treatment consideration because increased ambient pressure can reduce bubble volume and improve oxygen delivery [4,5]. However, its feasibility is dependent on local availability and transfer logistics. In this case, hyperbaric oxygen was not locally available; the nearest centre was more than one hour away, and no established emergency transfer pathway existed. Accordingly, hyperbaric treatment was not immediately available during the acute decision-making period.

Endovascular aspiration should not be considered routine treatment for cerebral air embolism. Successful aspiration of an intracranial air embolus causing middle cerebral artery occlusion has previously been reported outside the bronchoscopy setting [6]. In the present case, however, the radiological and histological findings support a mixed air-thrombotic large-vessel occlusion. Intravascular air can produce endothelial injury and thromboinflammatory responses, providing a plausible mechanism for secondary thrombosis [10]. A mixed air-thrombotic occlusion may therefore provide a potential target for aspiration in carefully selected patients with an accessible large-vessel occlusion, particularly when a thrombotic component is suspected. Nevertheless, this single case demonstrates technical feasibility rather than treatment efficacy and cannot determine the optimal sequencing of endovascular therapy and hyperbaric oxygen.

Complete angiographic reperfusion did not translate into substantial early neurological recovery. Despite TICI 3 reperfusion, the patient had persistent global aphasia, dysphagia, and dense right-sided weakness at 20 days and required inpatient stroke rehabilitation. Large-vessel recanalisation cannot reverse established cerebral infarction or address distal microembolisation and endothelial injury, which may explain the discrepancy between angiographic and clinical outcome. The practical implication is therefore not that thrombectomy should replace hyperbaric oxygen, but that endovascular assessment may be considered alongside hyperbaric consultation when imaging demonstrates a discrete, accessible large-vessel occlusion with a suspected thrombotic component. Institutions undertaking advanced bronchoscopic biopsy may consider predefined escalation pathways linking bronchoscopy, anaesthesia, stroke, neuroradiology, and hyperbaric services to facilitate rapid evaluation and treatment of suspected cerebral air embolism.

## Conclusions

Cerebral arterial air embolism should be considered when abrupt haemodynamic instability or unexplained delayed emergence follows bronchoscopic lung biopsy. Bradycardia and hypotension may provide an early clinical clue when they occur during or immediately after biopsy, although they are not specific for this complication. Once routine anaesthetic causes have been addressed, urgent non-contrast CT and CT angiography are important to identify intracranial air and determine whether a discrete large-vessel occlusion is present.

Hyperbaric oxygen remains an important treatment consideration, while endovascular aspiration may be considered as an adjunctive, time-critical option in carefully selected patients with an accessible large-vessel occlusion and a suspected thrombotic component. In this case, aspiration thrombectomy achieved TICI 3 reperfusion, but the patient had persistent global aphasia, dysphagia, and dense right-sided weakness at 20 days and required inpatient stroke rehabilitation. This case therefore demonstrates technical feasibility rather than treatment efficacy and highlights both the potential role and the limitations of large-vessel recanalisation in mixed air-thrombotic cerebral occlusion. 
